# The Effect of Microbiome-Derived Metabolites in Inflammation-Related Cancer Prevention and Treatment

**DOI:** 10.3390/biom15050688

**Published:** 2025-05-08

**Authors:** Alice N. Mafe, Dietrich Büsselberg

**Affiliations:** 1 Department of Biological Sciences, Faculty of Sciences, Taraba State University, Main Campus, Jalingo 660101, Taraba State, Nigeria; mafealice1991@gmail.com; 2 Weill Cornell Medicine-Qatar, Education City, Qatar Foundation, Doha Metropolitan Area, Al Rayyan P.O. Box 22104, Qatar

**Keywords:** microbiome metabolites, inflammation, cancer prevention, gut microbiota, immunomodulation

## Abstract

Chronic inflammation plays a crucial role in cancer development, yet the mechanisms linking the microbiome to inflammation-related carcinogenesis remain unclear. Emerging evidence suggests that microbiome-derived metabolites influence inflammatory pathways, presenting both challenges and opportunities for therapy. However, a deeper understanding of how these metabolites regulate inflammation and contribute to cancer prevention is still needed. This review explores recent advances in microbiome-derived metabolites and their roles in inflammation-related carcinogenesis. It highlights key molecular mechanisms, emerging therapies, and unresolved challenges. Synthesizing current research, including clinical trials and experimental models, bridges the gap between microbiome science and cancer therapy. Microbial metabolites such as short-chain fatty acids (SCFAs), polyamines, indoles, and bile acids play vital roles in regulating inflammation and suppressing cancer. Many metabolites exhibit potent anti-inflammatory and immunomodulatory effects, demonstrating therapeutic potential. Case studies show promising results, but challenges such as metabolite stability, bioavailability, and individual variability remain. Understanding microbiome–metabolite interactions offers novel strategies for cancer prevention and treatment. This review identifies knowledge gaps and proposes future research directions to harness microbiome-derived metabolites for innovative cancer therapies. Addressing these issues may pave the way for microbiome-targeted cancer interventions.

## 1. Introduction

Chronic inflammation is a well-established driver of cancer, contributing to tumor initiation, progression, and metastasis [1]. Recent evidence highlights the crucial role of the gut microbiome in modulating inflammation and tumorigenesis [2]. Among its many functions, the microbiome produces many metabolites that serve as critical signaling molecules, influencing immune responses, oxidative stress, and cellular pathways in cancer development [3]. These microbiome-derived metabolites have the potential to act as both pro- and anti-inflammatory agents, making them critical targets for cancer prevention and therapy [4]. Despite their growing recognition, the precise mechanisms through which these metabolites regulate inflammation and cancer risk remain incompletely understood [5]. Several gaps in knowledge and controversies hinder the full therapeutic utilization of microbiome-derived metabolites. The individual variability of the microbiome presents a challenge, as differences in microbial composition among individuals can significantly alter metabolite production and function [6]. Equally, the complex interplay in host–microbe interactions and the bioavailability of these metabolites in cancer therapy remains unclear [7]. While some metabolites exhibit promising anti-inflammatory and immunomodulatory effects, others may promote tumorigenesis under certain conditions, adding further complexity to their role in cancer biology [8]. Resolving these uncertainties is essential for developing effective microbiome-based interventions.

This review aims to synthesize recent discoveries, highlight emerging therapeutic strategies, and evaluate current obstacles in leveraging microbiome-derived metabolites for inflammation-related cancer prevention and treatment. This article provides insights into their clinical relevance and translational potential by critically assessing the molecular pathways and therapeutic innovations associated with these metabolites. Furthermore, this review explores the limitations of existing research, proposing novel interpretations and identifying core areas that require further investigation. The scope of this review encompasses the latest advancements in microbiome metabolite research, focusing on their role in modulating inflammation and their potential applications in cancer therapy. It critically evaluates preclinical and clinical findings, examines innovative therapeutic approaches, and discusses the broader implications of microbiome science in oncology. By tackling knowledge gaps and unresolved controversies, this study contributes to a more profound knowledge of microbiome–metabolite interactions and their significance in cancer prevention and treatment. This review comprehensively synthesizes microbiome–metabolite interactions in cancer, uncovering unexplored therapeutic potential and novel clinical applications. As research in this field progresses, realizing these interactions could pave the way for personalized microbiome-targeted strategies, offering innovative cancer prevention and treatment solutions.

## 2. Microbiome-Derived Metabolites and Their Role in Inflammation-Related Cancer

The gut microbiome produces a diverse range of metabolites that influence various physiological processes, including immune regulation, oxidative stress, and cellular signaling [9]. These microbial metabolites play a crucial role in modulating inflammation, which is an essential driver of cancer initiation and progression [10]. While some metabolites exhibit anti-inflammatory and tumor-suppressive effects, others contribute to carcinogenesis under specific conditions [11]. Perception of the functions of these significant microbial metabolites is essential for developing targeted strategies to mitigate inflammation-related cancer. Several studies have highlighted the impact of microbiome-derived metabolites on cancer progression (Table 1).

### 2.1. Major Microbial Metabolites and Their Functions

#### 2.1.1. Short-Chain Fatty Acids (SCFAs)

SCFAs, including butyrate, propionate, and acetate, are produced through bacterial fermentation of dietary fibers in the colon [23]. These metabolites are central to immune modulation and maintaining intestinal homeostasis [24]. Butyrate, in particular, has been extensively studied for its ability to induce apoptosis in cancer cells, regulate immune responses, and enhance gut barrier integrity [25]. Propionate and acetate also contribute to anti-inflammatory processes by modulating cytokine production and immune cell function [26]. The tumor-suppressive properties of SCFAs highlight their potential as therapeutic agents in cancer prevention and treatment [27].

#### 2.1.2. Polyamines

Polyamines, such as spermidine, are essential for cellular growth, differentiation, and immune regulation [28]. While polyamines are critical for normal physiological functions, dysregulated polyamine metabolism has been implicated in both inflammatory disorders and cancer [29]. Spermidine has shown promise in modulating inflammation by influencing autophagy, oxidative stress, and immune cell activity [30]. However, the dual role of polyamines in promoting both cell proliferation and apoptosis denotes the complexity of their involvement in cancer biology [31]. Further research is needed to harness the therapeutic potential of polyamine regulation in cancer treatment.

#### 2.1.3. Indoles and Tryptophan Metabolites

Indoles and tryptophan-derived metabolites, primarily produced by gut bacteria, play a significant role in modulating immune responses and inflammation [32]. These metabolites activate the aryl hydrocarbon receptor (AHR), a principal regulator of immune homeostasis and anti-inflammatory signaling [33]. By influencing T cell differentiation and cytokine production, indole derivatives contribute to immune tolerance and the suppression of chronic inflammation [34]. Their ability to counteract inflammatory pathways makes them potential candidates for microbiome-targeted cancer therapies [35].

#### 2.1.4. Bile Acid Derivatives

Secondary bile acids, produced through microbial metabolism of primary bile acids, exhibit diverse effects on immune regulation and tumorigenesis [36]. Some secondary bile acids, such as deoxycholic acid (DCA), have been linked to pro-inflammatory and tumor-promoting activities, while others, like lithocholic acid (LCA), exhibit immunosuppressive and anti-inflammatory properties [37]. The balance between these opposing effects is crucial in determining their role in cancer progression [38]. Discerning the interplay between bile acid metabolism and host immune responses is essential for designing microbiome-based interventions in cancer prevention [39]. The intricate relationship between microbiome-derived metabolites and inflammation-related cancer indicates the need for further investigation into their mechanisms of action [40]. By elucidating their molecular functions, researchers can develop innovative strategies to manipulate the microbiome for therapeutic benefits, offering new possibilities for cancer prevention and treatment [41].

#### 2.1.5. Lipopolysaccharide (LPS)—The Pro-Inflammatory Metabolite

Lipopolysaccharide (LPS), a key component of the outer membrane of Gram-negative bacteria, functions as a potent microbial-associated molecular pattern (MAMP) [42]. Under healthy conditions, LPS is typically confined to the gut lumen at low concentrations, where it contributes to the maturation and modulation of the immune system [43]. However, disruptions in the gut microbiota, which is referred to as dysbiosis, can compromise intestinal barrier integrity, leading to increased translocation of LPS into the bloodstream [44], as dysbiosis has been shown to increase LPS in circulation and leads to increased chronic inflammation, leading to a tumor-favoring environment; furthermore, it even acts molecularly to promote cancer. This rise in systemic LPS levels is known to trigger chronic low-grade inflammation, primarily through the activation of toll-like receptor 4 (TLR4), which stimulates the production of inflammatory mediators such as TNF-α, IL-6, and IL-1β [45]. Such sustained inflammation plays a pivotal role in creating a tumor-supportive microenvironment by promoting genomic instability, neovascularization, and immune suppression [46]. In addition to its immunostimulatory effects, LPS has been directly implicated in cancer progression. Recent findings demonstrate that in human cholangiocarcinoma, LPS enhances cancer cell migration and invasion by activating the METTL3/PI3K/AKT signaling axis, thereby contributing to tumor aggressiveness and metastatic potential [22]. Collectively, these insights emphasize the dualistic nature of microbial metabolites, with LPS exemplifying how dysbiosis-associated products can exacerbate inflammation-related carcinogenesis. Building on the earlier discussion of key microbial metabolites such as short-chain fatty acids (SCFAs), polyamines, and indoles, it is also essential to highlight the unique contributions of beneficial microbes like *Akkermansia muciniphila* and *Faecalibacterium prausnitzii*. These species are gaining recognition for their significant roles in inflammation regulation and cancer prevention. They are increasingly considered next-generation probiotics due to their strong anti-inflammatory and immunoregulatory capabilities [47]. *A. muciniphila* is notable for its ability to degrade mucin, strengthen gut barrier function, and downregulate pro-inflammatory cytokines, thereby potentially inhibiting tumor initiation and progression [48]. In contrast, *F*. *prausnitzii* is a key butyrate producer and is consistently associated with reduced incidence of inflammatory bowel disease and colorectal cancer [49]. Compared with traditional probiotics such as *Bifidobacteria* and *Lactobacilli*, which are primarily known for promoting overall gut health and supporting immune function, *A. muciniphila* and *F. prausnitzii* offer more specialized effects by modulating host signaling pathways linked to inflammation and carcinogenesis. Their ability to influence pathways such as NF-κB, IL-10, and Treg cell responses highlights their potential as therapeutic agents in inflammation-associated cancers [50].

## 3. Molecular Mechanisms Linking Microbiome Metabolites to Cancer Prevention

**Figure 1:**
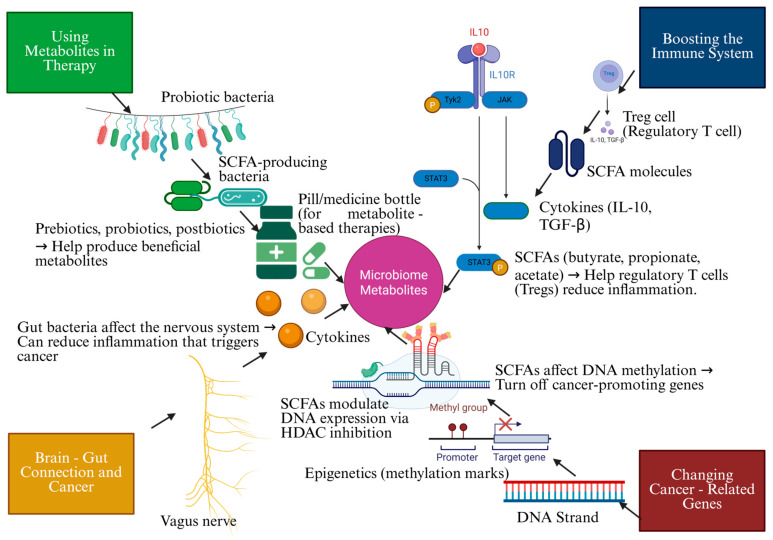
Microbiome metabolites: essential mechanisms in cancer prevention and therapy (created with BioRender).

**Figure 2:**
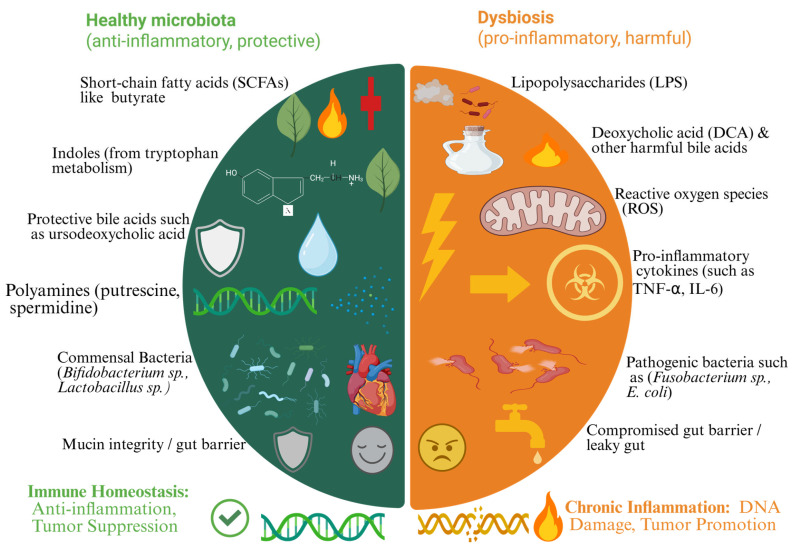
The double-edged sword of the gut microbiome: comparison of healthy microbiota and dysbiosis in cancer prevention and promotion (created with BioRender).

Microbiome-derived metabolites exert profound effects on cancer prevention through multiple molecular pathways. These metabolites influence immune responses, epigenetic modifications, and systemic signaling networks, all of which play critical roles in tumor suppression [51]. Microbial metabolites can promote or inhibit cancer progression by modulating inflammation, metabolic reprogramming, and neuroimmune interactions [52]. Knowing these molecular mechanisms provides new opportunities for harnessing the microbiome as a therapeutic target in cancer prevention [53]. Figure 1 illustrates the protective role of microbiome metabolites in cancer prevention and therapy. As illustrated in Figure 2, the gut microbiome plays a dual role in cancer prevention and promotion depending on its composition.

### 3.1. Immunomodulation and Anti-Inflammatory Pathways

The interplay between microbiome-derived metabolites and immune signaling pathways plays a crucial role in modulating inflammation and promoting anticancer effects [54].

#### 3.1.1. SCFAs and Regulatory T Cell Activation

Short-chain fatty acids (SCFAs), particularly butyrate, propionate, and acetate, are central players in immune regulation [55]. These microbial metabolites promote the differentiation and activation of regulatory T cells (Tregs) [56], which are essential for maintaining immune tolerance and suppressing chronic inflammation [57]. SCFAs enhance Treg function by modulating histone deacetylase (HDAC) activity and increasing the production of anti-inflammatory cytokines such as IL-10 [58]. SCFAs promote an immunosuppressive environment, helping counteract the pro-inflammatory conditions that drive cancer development. Thus, they are crucial mediators of cancer prevention.

#### 3.1.2. Gut Microbiota and Tumor Microenvironment Modulation

The gut microbiota plays a fundamental role in shaping the tumor microenvironment by influencing immune cell recruitment, cytokine signaling, and metabolic interactions [59]. Microbiome-derived metabolites regulate the balance between pro- and anti-inflammatory factors within the tumor microenvironment, thereby affecting cancer progression [60]. For instance, certain bacterial species produce metabolites that activate immune surveillance mechanisms, enhancing the cytotoxic activity of natural killer (NK) cells and cytotoxic T lymphocytes [61]. Short-chain fatty acids (SCFAs), important microbial metabolites, influence gene regulation not by directly binding to DNA but by inhibiting histone deacetylases (HDACs), which leads to changes in chromatin structure and subsequently affects gene expression [62]. Conversely, dysbiosis or an imbalance in microbiome composition can lead to an inflammatory tumor-promoting environment, underscoring the importance of microbiome stability in cancer prevention [63].

### 3.2. Epigenetic and Metabolic Reprogramming

#### 3.2.1. Microbiome Influence on Oncogene Expression and DNA Methylation

Microbiome metabolites directly impact gene expression by modulating oncogenes and tumor suppressor genes through epigenetic mechanisms [64]. For example, SCFAs such as butyrate act as HDAC inhibitors, altering chromatin structure and influencing transcriptional regulation [65]. Moreover, microbial metabolites can affect DNA methylation patterns, leading to changes in gene expression that either promote or inhibit tumorigenesis [66]. These epigenetic modifications are crucial in regulating inflammation and cellular transformation, positioning microbiome metabolites as leading players in cancer prevention.

#### 3.2.2. Metabolite-Driven Epigenetic Changes in Inflammation

Beyond oncogene regulation, microbiome metabolites also induce epigenetic modifications in inflammatory pathways [67]. For instance, indole derivatives and polyamines influence histone modifications and DNA methylation patterns associated with inflammation and immune responses [68]. By suppressing pro-inflammatory gene expression and enhancing anti-inflammatory pathways, these metabolites contribute to a protective environment against chronic inflammation-driven cancers [69]. Cognizance of these epigenetic interactions opens new possibilities for developing microbiome-targeted interventions for cancer prevention.

### 3.3. Gut–Brain Axis and Systemic Effects on Cancer

#### Neuroimmune Interactions and Inflammatory Signaling Pathways

The gut microbiome communicates with the central nervous system via the gut–brain axis, influencing systemic inflammation and immune responses [70]. Neurotransmitters and microbial metabolites such as serotonin, dopamine, and SCFAs regulate neuroimmune interactions, impacting inflammatory signaling pathways associated with cancer progression [71]. Chronic stress and dysregulated neuroimmune signaling can exacerbate inflammation and promote tumor growth, while microbiome-derived metabolites can counteract these effects by modulating neuroinflammatory pathways [72]. This highlights the systemic influence of the microbiome on cancer prevention, emphasizing the need for a holistic approach to microbiome research in oncology. By elucidating these molecular mechanisms, researchers can identify new strategies to manipulate microbiome-derived metabolites for cancer prevention. Targeting these pathways through dietary interventions, probiotics, or microbiome-modulating therapies holds significant promise for reducing cancer risk and improving long-term health outcomes.

Beyond the immunomodulatory functions of metabolites like short-chain fatty acids (SCFAs), certain gut microbes, particularly *Akkermansia muciniphila* and *Faecalibacterium prausnitzii*, exert direct regulatory effects on the immune system that are crucial for cancer prevention. *A. muciniphila* contributes to gut barrier integrity by promoting mucus layer regeneration and reinforcing tight junctions, thereby limiting the entry of pro-inflammatory molecules and microbial products into systemic circulation, a process implicated in tumor development [73]. It also supports immune balance by inducing anti-inflammatory cytokines such as IL-10 and modulating dendritic cell function [74]. Likewise, F. prausnitzii, through its production of butyrate and other bioactive compounds, boosts the expansion of regulatory T cells (Tregs) while inhibiting pro-inflammatory cytokines like IL-6 and TNF-α, contributing to an anti-inflammatory and anti-tumor environment [75]. Compared with traditional probiotics such as *Bifidobacteria* and *Lactobacilli*, which primarily enhance general immune health and gut microbial balance, *A. muciniphila* and *F. prausnitzii* offer more specialized effects by targeting immune pathways involved in inflammation and cancer progression [76]. These unique immunological interactions highlight their potential as next-generation probiotics for cancer prevention.

## 4. Microbiome Metabolites in Cancer Therapy: Innovations and Emerging Strategies

Advancements in microbiome research have paved the way for innovative cancer therapies that leverage microbial metabolites to modulate inflammation, immune responses, and tumor progression [77]. Emerging strategies focus on harnessing the therapeutic potential of microbiome-derived metabolites through prebiotic- and probiotic-based approaches, metabolite supplementation, microbiome engineering, and metabolite-targeted drug development [78]. These interventions offer promising avenues for enhancing cancer prevention and treatment while attending to the setbacks associated with microbiome variability and metabolite bioavailability.

### 4.1. Prebiotic- and Probiotic-Based Therapies

Prebiotic- and probiotic-based therapies aim to modulate the gut microbiome composition to enhance the production of beneficial metabolites with anti-inflammatory and anticancer properties [79]. Prebiotics, such as dietary fibers and polyphenols, serve as substrates for beneficial gut bacteria, promoting the production of short-chain fatty acids (SCFAs) and other bioactive compounds [80]. On the other hand, probiotics introduce live beneficial microorganisms, such as *Lactobacillus* sp. and *Bifidobacterium* species, which can enhance gut health and contribute to immune regulation [81]. Clinical studies suggest that probiotic supplementation can improve responses to cancer therapies, reduce inflammation, and enhance immune checkpoint inhibitor efficacy [82]. Capsule-based delivery systems have been designed to enhance the stability and targeted gut release of SCFAs, preventing their degradation before reaching the site of action [83]. Moreover, emerging approaches focus on engineering probiotic strains to produce and release SCFAs directly within the gastrointestinal tract, amplifying their localized anti-inflammatory and anticancer effects [84]. However, further research is needed to personalize prebiotic and probiotic interventions based on individual microbiome profiles for optimal therapeutic outcomes. Table 2 outlines recent advancements in utilizing microbiome metabolites for cancer therapy.

### 4.2. Postbiotics and Metabolite Supplementation

Postbiotics, which refer to bioactive compounds derived from microbial metabolism, offer a promising alternative to live probiotics [95]. These include SCFAs, polyamines, indoles, and other microbial metabolites with immunomodulatory and tumor-suppressive effects [96]. Direct supplementation with these metabolites bypasses problems associated with probiotic colonization and microbiome variability, providing a more controlled therapeutic approach [97]. Butyrate, for instance, has been investigated for its ability to induce apoptosis in cancer cells and improve gut barrier integrity [98]. Similarly, indole derivatives have demonstrated anti-inflammatory properties that could be leveraged in cancer therapy [99]. The development of metabolite-based supplements holds great potential, but setbacks related to bioavailability, stability, and targeted delivery need to be addressed for clinical application.

### 4.3. Synthetic Microbiome Engineering for Precision Medicine

Synthetic microbiome engineering is an emerging field that aims to design and modify bacterial strains for therapeutic applications [100]. By genetically engineering bacteria to produce specific anticancer metabolites, researchers can develop precision microbiome-based interventions tailored to individual patients [101]. Engineered probiotics, for example, can be programmed to secrete SCFAs, polyamines, or bile acid derivatives that regulate inflammation and immune responses within the tumor microenvironment [102]. Also, synthetic microbiomes can enhance the efficacy of existing cancer therapies, such as immunotherapy and chemotherapy, by modulating drug metabolism and immune checkpoint activity [103]. While this approach holds great promise, trials must be carefully considered before clinical translation, and factors such as microbial stability, regulatory approvals, and potential off-target effects must be considered.

### 4.4. Metabolite-Targeted Drug Development

Metabolite-targeted drug development seeks to harness microbiome-derived metabolites as therapeutic agents or develop synthetic analogs that mimic their beneficial effects [104]. Small-molecule drugs inspired by microbial metabolites are being explored for their ability to modulate epigenetic, immune, and metabolic pathways involved in cancer progression [105]. For example, HDAC inhibitors based on SCFAs are being developed to regulate gene expression and suppress tumor growth [106]. In addition, bile acid analogs and indole-based compounds are being investigated for their ability to target inflammation-driven cancers [107]. The integration of microbiome-derived metabolites into drug discovery pipelines offers a novel approach to developing next-generation cancer therapeutics with enhanced efficacy and specificity [108]. The application of microbiome metabolites in cancer therapy represents a rapidly evolving field with immense therapeutic potential [109]. By integrating microbiome science with precision medicine, researchers can develop innovative interventions that target inflammation-related cancer mechanisms [110]. Continued exploration of these emerging strategies will be crucial for translating microbiome-based therapies into effective clinical treatments for cancer prevention and management.

## 5. Case Studies and Controversies in Microbiome Metabolite Research

Despite growing interest in the role of microbiome-derived metabolites in cancer prevention and therapy, significant controversies and roadblocks remain [111]. Clinical studies have demonstrated promising and conflicting results, featuring the complexity of microbiome–metabolite interactions in cancer [112]. While some trials support the protective effects of microbial metabolites, others reveal limitations, including inter-individual variability, metabolite stability, and inconsistent therapeutic outcomes [113]. This section explores pivotal case studies and unresolved debates surrounding microbiome metabolites in cancer research.

### 5.1. Clinical Trials on SCFAs in Colorectal Cancer Prevention

Short-chain fatty acids (SCFAs), particularly butyrate, have been extensively studied for their potential in colorectal cancer prevention [114]. Several clinical trials have investigated the role of dietary fiber supplementation in increasing SCFA production and reducing colorectal cancer risk [115]. Butyrate has been shown to promote apoptosis in cancer cells, enhance gut barrier integrity, and modulate immune responses [116]. Studies suggest that individuals with higher SCFA levels exhibit reduced inflammation and a lower incidence of colorectal cancer [117]. However, not all clinical trials have produced consistent results. Some studies indicate that while SCFAs exhibit anti-inflammatory and tumor-suppressive properties in early-stage cancer, they may promote tumor growth under specific conditions, such as hypoxic tumor microenvironments [118]. Likewise, individual gut microbiota composition differences may influence SCFA production, leading to variable outcomes [119]. These findings mark the need for personalized approaches to microbiome-based cancer prevention strategies. Table 3 summarizes vital case studies and ongoing controversies in microbiome metabolite research.

### 5.2. Challenges and Controversies in Metabolite-Based Cancer Therapy

Microbiome-derived metabolites, such as polyamines and SCFAs, have emerged as promising candidates in oncology. However, their clinical application remains challenging due to conflicting evidence and biological complexities. Among them, polyamines like spermidine and putrescine exhibit a paradoxical role in cancer progression [130]. Animal studies have shown that dietary spermidine promotes autophagy, enhances mitochondrial function, and reduces oxidative stress. For example, Yue et al. (2017)demonstrated that spermidine supplementation extended lifespan and suppressed hepatocellular carcinoma in mice via autophagy-related gene activation [131]. Likewise, Gerner et al. (2018) studied APCMin/+ mice, a model for colorectal cancer, and found that spermidine reduced tumor burden and enhanced immune surveillance through activation of cytotoxic T cells [132]. Conversely, other studies suggest that elevated polyamine levels can drive tumorigenesis, especially in advanced cancers [133]. A Phase I/II trial of polyamine blockade therapy (PBT) using difluoromethylornithine (DFMO) and sulindac in metastatic colorectal cancer showed reduced adenoma recurrence but limited efficacy in late-stage disease due to compensatory polyamine uptake [134]. Moreover, high expression of ornithine decarboxylase (ODC), the rate-limiting enzyme in polyamine synthesis, has been linked to poor prognosis in breast and prostate cancers. These contradictory findings have led to debates about the appropriateness of polyamine-targeted interventions, emphasizing the need for biomarker-driven, personalized approaches [135].

Beyond polyamines, other microbial metabolites, such as SCFAs (e.g., butyrate and propionate), indoles, and secondary bile acids, also show protective and adverse effects. Butyrate, a known histone deacetylase (HDAC) inhibitor with anti-inflammatory properties, has demonstrated anticancer potential. In a mouse model, dietary inulin increased colonic butyrate levels, reducing tumor formation and promoting apoptosis [136]. Yet, under hypoxic conditions, butyrate may serve as an energy source for cancer cells, possibly supporting tumor growth depending on the tumor microenvironment. The therapeutic use of these metabolites is further limited by their instability and short systemic half-lives [137]. SCFAs and indole derivatives are rapidly metabolized in the gut, hindering their delivery to distal tumors. For instance, oral sodium butyrate showed local benefits in ulcerative colitis but failed to achieve sufficient plasma levels for treating extraintestinal malignancies [138]. Inter-individual differences in gut microbiota composition also influence metabolite production and treatment outcomes. A recent Phase II trial of *Akkermansia muciniphila* as an immunotherapy adjuvant found that responders had gut profiles enriched in SCFAs and indole-3-lactic acid. At the same time, non-responders lacked these metabolites despite receiving the same probiotic [139]. These findings underscore the need for precision microbiome strategies based on individual microbial, dietary, and genetic factors.

Another major challenge is the lack of standardized dosing protocols for metabolite-based therapies. Unlike conventional drugs, these compounds lack well-defined pharmacokinetic and pharmacodynamic data. In a Phase I trial, indole-3-carbinol (a dietary precursor of indole metabolites) showed dose-related effects on estrogen metabolism and immunity in women with cervical intraepithelial neoplasia, but inconsistent microbial conversion led to varied responses [140]. Safety concerns also persist. Though generally considered safe, long-term use of probiotics, prebiotics, and postbiotics can disrupt gut microbial balance. Rare cases of bacteremia and metabolic disturbances have been reported in immunocompromised individuals following high-dose probiotic use. Furthermore, interactions between microbial metabolites and anticancer drugs may lead to unintended immune or metabolic effects [141]. While microbiome-derived metabolites represent an exciting frontier in cancer prevention and therapy, their clinical integration faces multiple hurdles, including mechanistic ambiguities, delivery barriers, patient variability, and safety concerns. Future progress will depend on well-designed clinical trials, the identification of reliable biomarkers, and tailored interventions aligned with individual microbiota and disease profiles [142].

## 6. Complications, Limitations, and Future Perspectives

Despite the promising potential of microbiome-derived metabolites in cancer prevention and therapy, several issues must be addressed before these strategies can be widely implemented in clinical settings [143]. Variability in individual microbiomes, limitations in metabolite bioavailability, and regulatory concerns present significant hurdles [144]. However, emerging research directions, including personalized microbiome-based therapies and integrative multi-omics approaches, offer opportunities to overcome these obstacles and enhance the therapeutic potential of microbiome metabolites in oncology [145].

### 6.1. Complications and Limitations

Despite significant progress in microbiome metabolite research, several hurdles and limitations persist, including variability in individual microbiomes, mechanistic complexities, and translational hurdles in clinical applications [146]. Table 4 outlines the chief difficulties, limitations, and future directions in microbiome metabolite research.

#### 6.1.1. Variability in Individual Microbiomes

One of the biggest trials in microbiome-based cancer therapies is the high degree of variability in microbiome composition among individuals [152]. Factors such as genetics, diet, lifestyle, and antibiotic exposure influence the gut microbiota, leading to differences in metabolite production and therapeutic responses [153]. This variability makes it difficult to develop universal microbiome-targeted interventions [154]. Personalized approaches that consider individual microbiome profiles are necessary to maximize therapeutic efficacy and minimize unintended effects [155].

#### 6.1.2. Bioavailability and Stability of Metabolites

Many microbiome-derived metabolites, such as SCFAs and indole derivatives, have limited stability and bioavailability, reducing their effectiveness as therapeutic agents [156]. These metabolites are often rapidly metabolized or degraded in the gut before they can exert systemic effects [157]. Developing novel drug delivery systems, such as encapsulated formulations or targeted release mechanisms, could enhance metabolite stability and improve their therapeutic impact [158]. Further research is needed to optimize metabolite-based interventions for clinical application.

#### 6.1.3. Regulatory and Ethical Considerations

Regulatory approval for microbiome-based therapies presents another significant challenge. The classification of probiotics, prebiotics, and postbiotics as dietary supplements rather than pharmaceutical drugs [159] complicates the regulatory process for their clinical use in cancer treatment [160]. Standardized guidelines for quality control, safety, and efficacy are required to ensure the consistency and reliability of microbiome-based interventions [161]. In the same vein, ethical concerns related to microbiome manipulation, such as unintended long-term effects and potential microbiome engineering risks, must be carefully evaluated before these therapies can be widely adopted [162].

### 6.2. Future Research Directions

#### 6.2.1. Personalized Microbiome-Based Therapies

To overcome microbiome variability, future research should focus on personalized approaches that tailor microbiome-based therapies to individual patients [163]. Advances in microbiome sequencing and computational modeling can help identify specific microbial signatures associated with cancer risk and therapeutic response [164]. Personalized interventions, such as customized probiotics, targeted prebiotics, or patient-specific metabolite supplementation, could improve treatment outcomes while minimizing adverse effects.

#### 6.2.2. Advanced Multi-Omics Approaches for Precision Medicine

Integrating multi-omics technologies including metagenomics, metabolomics, transcriptomics, and proteomics will provide a more comprehensive interpretation of microbiome–metabolite interactions in cancer [165]. By combining these datasets, researchers can uncover novel biomarkers, predict therapeutic responses, and develop more effective microbiome-targeted interventions [166]. Machine learning and artificial intelligence (AI) will play a crucial role in analyzing complex microbiome data and identifying optimal therapeutic strategies [167].

#### 6.2.3. Integration of Microbiome Metabolites with Immunotherapy and Chemotherapy

Another promising research direction is the integration of microbiome metabolites with existing cancer treatments, such as immunotherapy and chemotherapy [168]. Microbiome-derived metabolites have been shown to enhance immune checkpoint blockade therapy, modulate the tumor microenvironment, and reduce treatment-related side effects [169]. Future studies should explore how microbial metabolites can be combined with conventional cancer therapies to improve patient outcomes [170]. Additionally, clinical trials investigating microbiome metabolite-based adjuvants will be essential for translating these findings into practical cancer treatment strategies [171]. As microbiome research advances, treating these problems and embracing emerging research directions will be crucial for harnessing the full therapeutic potential of microbiome metabolites in cancer prevention and treatment. By integrating personalized medicine, innovative drug delivery systems, and precision oncology approaches, microbiome-based interventions may become a transformative strategy in the fight against cancer.

## 7. Conclusions

The role of microbiome-derived metabolites in cancer prevention and treatment has gained significant attention, spotlighting their potential as essential modulators of inflammation and tumor progression. This review has explored the diverse functions of microbial metabolites, such as short-chain fatty acids (SCFAs), polyamines, and bile acid derivatives, in regulating immune responses, epigenetic modifications, and metabolic pathways associated with cancer. While promising preclinical and clinical studies suggest the therapeutic value of microbiome-based interventions, trials related to bioavailability, metabolism, and individual variability must be addressed to optimize their clinical application. Future research should focus on developing personalized microbiome-based therapies that consider individual variations in gut microbiota composition and metabolite production. Advanced drug delivery strategies, such as encapsulation and targeted release mechanisms, could enhance the stability and bioactivity of microbial metabolites, improving their therapeutic efficacy. Furthermore, integrating microbiome-derived interventions with conventional cancer treatments, such as immunotherapy and chemotherapy, presents a novel avenue for enhancing treatment responses and reducing adverse effects. As our grasp of microbiome–metabolite interactions continues to evolve, translating these findings into clinical applications will require interdisciplinary collaboration, rigorous clinical trials, and the development of regulatory frameworks for microbiome-based therapeutics. By managing current issues and leveraging emerging innovations, microbiome-derived metabolites have the potential to revolutionize cancer prevention and treatment strategies, paving the way for more effective and personalized approaches in oncology.

## Figures and Tables

**Table 1:** Microbiome-derived metabolites and their role in inflammation-related cancer.

Major Findings	Metabolites Studied	Cancer Type
Butyrate induces apoptosis	SCFAs	Colorectal [12]
Propionate suppresses inflammation	SCFAs	Liver [13]
Spermidine regulates tumor growth	Polyamines	Breast [14]
Indoles modulate immune responses	Indole derivatives	Prostate [15]
Bile acids impact gut microbiota	Secondary bile acids	Pancreatic [16]
SCFAs enhance gut barrier function	SCFAs	Gastric [17]
Microbiome-derived metabolites impact metastasis	Various	Lung [18]
Indole derivatives affect inflammatory markers	Indoles	Ovarian [19]
Polyamine levels correlate with tumor progression	Polyamines	Multiple [20]
SCFAs modulate T cell differentiation	SCFAs	Colorectal [21]
LPS promotes chronic inflammation and tumor progression via TLR4 and METTL3/PI3K/AKT signaling	LPS	Cholangiocarcinoma [22]

**Table 2:** Microbiome metabolites in cancer therapy: innovations and emerging strategies.

Approach	Target	Cancer Type
Probiotic supplementation	Gut microbiota balance	Colorectal [85]
Prebiotic fiber therapy	SCFA production	Liver [86]
SCFA-based treatment	Tumor suppression	Gastric [87]
Engineered microbiome therapies	Precision medicine	Breast [88]
Postbiotic metabolites as drugs	Anti-inflammatory effects	Ovarian [89]
Microbiome modification	Tumor microenvironment	Pancreatic [90]
Metabolite-targeted drugs	Immune modulation	Lung [91]
Synthetic microbiota engineering	Personalized therapy	Multiple [92]
Bile acid modulation	Tumor immunotherapy	Liver [93]
SCFAs combined with immunotherapy	Combination therapy	Colorectal [94]

**Table 3:** Case studies and controversies in microbiome metabolite research.

Trial Phase	Findings
Phase I	SCFAs reduced inflammation [120]
Phase II	Butyrate improved immune response [121]
Phase III	Mixed results on tumor regression [122]
Phase I	SCFA bioavailability drawbacks [123]
Phase II	Combination therapy with SCFAs [124]
Phase III	SCFA-based diets showed limited efficacy [125]
Phase II	Butyrate supplementation improved outcomes [126]
Phase III	No significant tumor regression observed [127]
Phase II	SCFAs enhanced chemotherapy response [128]
Phase III	SCFAs reduced side effects of treatment [129]

**Table 4:** Complications, limitations, and future perspectives.

Complication	Findings
Microbiome variability	Microbiome composition differs across populations [147]
Bioavailability issues	SCFAs degrade quickly in circulation [148]
Ethical concerns	Engineering gut microbiota raises safety issues [149]
Regulatory barriers	Lack of FDA-approved microbiome therapies [150]
Individual response variability	Different patients respond differently to SCFAs [151]

## Data Availability

Not applicable.

## Abbreviations

SCFAs	Short-chain fatty acids
AHR	Aryl hydrocarbon receptor
LCA	Lithocholic acid
Tregs	Regulatory T cells
HDAC	Histone deacetylase
NK	Natural killer
AI	Artificial intelligence
